# ^123^I-mIBG scintigraphy in neuroblastoma: development of a SIOPEN semi-quantitative reporting ,method by an international panel

**DOI:** 10.1007/s00259-016-3516-0

**Published:** 2016-09-24

**Authors:** V. Lewington, B. Lambert, U. Poetschger, Z. Bar Sever, F. Giammarile, A. J. B. McEwan, Rita Castellani, T. Lynch, B. Shulkin, M. Drobics, A. Staudenherz, R. Ladenstein

**Affiliations:** 1 King’s College, London, UK; 2 Radiology and Nuclear Medicine, Ghent University, Ghent, Belgium; 3 Department for Studies and Statistics on Integrated Research and Projects (S2IRP), Children’s Cancer Research Institute, Vienna, Austria; 4 Schneider Children’s Medical Centre of Israel, Petach-Tikva, Israel; 5 CHLS, Pierre-Benite, France; 6 Cross Cancer Institute, Edmonton, Canada; 7 Nuclear Medicine, Fondazione IRCCS Istituto Nazionale dei Tumori, Milan, Italy; 8 Northern Ireland Cancer Centre, Belfast, UK; 9 St Jude’s Children’s Research Hospital, Memphis, USA; 10 AIT Austrian Institute of Technology GmbH Safety & Security Department, Information Management & eHealth, Vienna, Austria; 11 AKH, Vienna, Austria; 12 St. Anna Children’s Hospital and Medical University, Vienna, Austria

**Keywords:** Neuroblastoma, 123-meta-iodobenzylguanidine [123I-mIBG], Imaging, Scintigraphy, Skeleton, SIOPEN-R-NET

## Abstract

**Purpose:**

A robust method is required to standardise objective reporting of diagnostic ^123^I-mIBG images in neuroblastoma. Prerequisites for an appropriate system are low inter- and intra-observer error and reproducibility across a broad disease spectrum. We present a new reporting method, developed and tested for SIOPEN by an international expert panel.

**Method:**

Patterns of abnormal skeletal ^123^I-mIBG uptake were defined and assigned numerical scores [0–6] based on disease extent within 12 body segments. Uptake intensity was excluded from the analysis. Data sets from 82 patients were scored independently by six experienced specialists as unblinded pairs (pre- and post-induction chemotherapy) and in random order as a blinded study. Response was defined as ≥50 % reduction in post induction score compared with baseline.

**Results:**

In total, 1968 image sets were reviewed individually. Response rates of 88 % and 82 % were recorded for patients with baseline skeletal scores ≤23 and 24-48 respectively, compared with 44 % response in patients with skeletal scores >48 (*p* = 0.02). Reducing the number of segments or extension scale had a small but statistically negative impact upon the number of responses detected. Intraclass correlation coefficients [ICCs] calculated for the unblinded and blinded study were 0.95 at diagnosis and 0.98 and 0.99 post-induction chemotherapy, respectively.

**Conclusions:**

The SIOPEN mIBG score method is reproducible across the full spectrum of disease in high risk neuroblastoma. Numerical assessment of skeletal disease extent avoids subjective evaluation of uptake intensity. This robust approach provides a reliable means with which to examine the role of 123I mIBG scintigraphy as a prognostic indicator in neuroblastoma.

## Introduction

Whole-body imaging using radiolabelled meta-iodobenzylguanidine [mIBG] has an established place in staging and monitoring treatment response in neuroblastoma [NB] [1, 2]. In addition to descriptive image reporting, a standardised, objective evaluation method is required to compare patient populations and treatment responses in multi-centre clinical trials. Pre-requisites for a suitable scoring system are i. reproducibility, ii. validity across the full spectrum of disease, and iii. straightforward application by trained specialists with minimal requirement for additional training.

Several semi-quantitative mIBG reporting methods have been described previously, based upon the allocation of numerical scores to document mIBG positive disease extent and intensity of mIBG uptake compared with reference normal tissues [3–6]. In contrast to positron emission tomography [PET] imaging, where standardised uptake value (SUV) measurements are well established [7, 8] visual assessment of mIBG uptake intensity is subjective and highly variable. Variability could be reduced by focusing on disease extent rather than uptake intensity in individual lesions. A new, semi-quantitative objective reporting method based on this principle has been developed and tested by an international panel of seven experienced nuclear medicine specialists.

## Method

**Fig. 1:**
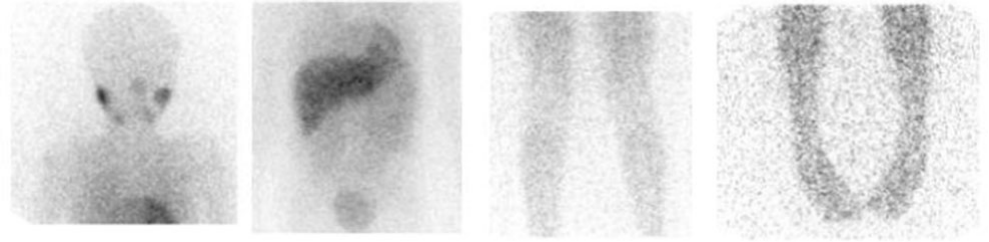
Normal anterior ^123^I-mIBG images showing physiological salivary, myocardial, and hepatic uptake and excreted activity in the urinary bladder. Skeletal Score = 0

**Fig. 2:**
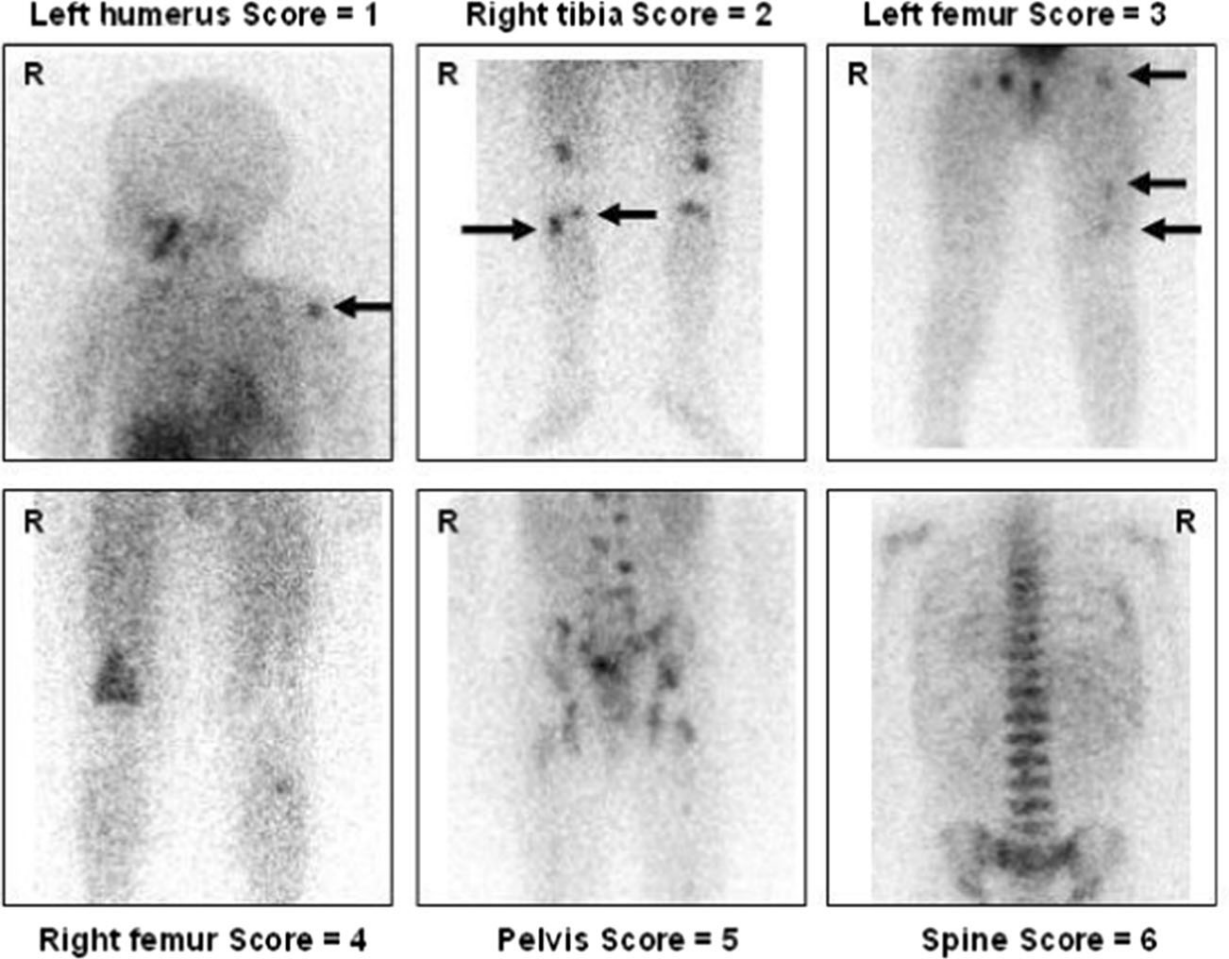
^123^I-mIBG images illustrating abnormal skeletal uptake corresponding to scores 1–6. Top row: individual lesions are arrowed. Bottom row: patterns of diffuse skeletal uptake

The skeletal distribution of mIBG was recorded in 12 anatomical body segments (Table 1) as follows: skull, thoracic cage, proximal right upper limb, distal right upper limb, proximal left upper limb, distal left upper limb, spine, pelvis, proximal right lower limb, distal right lower limb, proximal left lower limb, and distal left lower limb. The extent and pattern of skeletal mIBG involvement were scored using a 0–6 scale to discriminate between focal discrete lesions and patterns of more diffuse infiltration as described in Table 2. Examples of the different extension scores are given in Figs. 1 and 2.

**Table 1:** 12 segment SIOPEN skeletal score versus a comparator 7 segment score

12 segment method	7 segment method
Skull	Skull
Right humerus	Upper limbs
Left humerus	
Right radius/ulna	
Left radius/ulna	
Thoracic cage	Thoracic cage
Spine	Spine
Pelvis	Pelvis
Right femur	Femora
Left femur	
Right tibia/fibula	Distal lower limbs
Left tibia/fibula

**Table 2:** SIOPEN skeletal score 0–6 extension scale versus comparator 0-3 scale

^123^ I-mIBG skeletal extension scale 0–6		Extension scale 0–3	
Skeletal score	Descriptor	Score	Descriptor
0	No abnormality	0	No abnormality
1	1 discrete focus	1	Solitary abnormality
2	2 discrete foci	2	>1 abnormality
3	3 discrete foci		
4	>3 discrete foci or diffuse involvement < 50 % bone	3	Diffuse (>50 % of segment involved)
5	Diffuse involvement >50–95 % whole bone		
6	Uniform, diffuse whole bone involvement

Primary tumours and soft tissue disease were evaluated separately, focusing on lesion size relative to normal myocardium.

This method was applied to anonymised mIBG image sets performed in children with NB uniformly treated within the SIOPEN-R-NET High Risk NB Study. Parental written informed consent was obtained at trial entry. All imaging procedures were undertaken using iodine I-123 (^123^I) radio-labelled mIBG in accordance with local hospital acquisition protocols based upon published European guidelines [9]. Imaging was performed at diagnosis and on completion of rapid COJEC induction chemotherapy [10]. Image data from participating European centres were stored electronically on the SIOPEN-R-NET database.

For the purpose of the review, the minimum image quality standard was defined as whole-body anterior and posterior scans or overlapping anterior and posterior static images including all peripheries, acquired within 18-24 h of intravenous tracer ^123^I-mIBG injection. JPEG, TIFF, and DICOM image formats were included. As few centres undertake paediatric single photon emission tomography (SPECT) routinely, SPECT data were omitted from the analysis. No fused SPECT/CT data were reviewed. All mIBG images held on the SIOPEN-R-NET database were considered. Eighty-two patients met the review criteria of two complete, high quality data sets obtained at diagnosis and on completion of rapid COJEC induction chemotherapy. The number of individual images contained within each data set varied, depending on whether whole body or overlapping static views were acquired.

Images were scored independently by six nuclear medicine specialist panel members as pre- and post-treatment image pairs (“unblinded”) and again as individual scans in random order as a blinded study. Three hundred and twenty-eight separate data sets were reviewed by each panel member, and 1968 complete reviews were performed. File headers indicated the primary tumour site, but no further clinical information or correlative imaging was made available.

The 12-segment method used does not map exactly to mIBG scoring systems reported previously [3–6]. To assess whether the method could be simplified by reducing the number of surveyed segments, results were compared with a hypothetical seven-segment model derived from earlier published methods [3–6]. (Table 1). The 0–6 scale demonstrating skeletal disease extent was compared with a 0–3 scale described previously (Table 2) [3, 5].

Changes in primary and soft tissue tumour size and uptake pattern were assessed qualitatively from post-treatment images in the unblinded study.

Interclass correlation coefficients were used to assess intra- and inter-observer variability. The frequency of skeletal metastasis by anatomical site and relative contribution of different anatomical regions to overall score were calculated.

The typical time to score a downloaded, complete image set was noted.

### Statistical method

Skeletal mIBG scores were compared at diagnosis and after rapid COJEC induction chemotherapy using Spearman correlation coefficients. Spearman Rank order correlation is a non-parametric measure of association based on the ranks of data values between−1 and 1. A positive relationship exists if the correlation is greater than 0. Bland Altman plots [11] were used to evaluate agreement between the new score method and alternative systems, comparing 0–6 vs. 0–3 score scales and 12 vs. 7 body segment scores respectively. The Bland–Altman plot displays the difference between methods against their mean.

The maximal score of 72 was divided in thirds to categorize patients at diagnosis in low (score 0–23), intermediate (24–48), and high (49–72) skeletal scores. Response to induction chemotherapy (defined as ≥50 % score decrease) was compared for each group using the chi squared test.

Intraclass correlation coefficients (ICC) were used to assess the reliability between multiple observers [12]. ICC is defined as the ratio of the between-patient variance to the total variance, which is a combination of inter-patient and intra- patient variance (i.e. variance between observers) and estimated using a random effect model.

## Results

1968 mIBG data sets from 34 participating SIOPEN-R-NET centres in 12 European countries were reviewed. An equal number of images was reviewed in DICOM format and as JPEG/TIFF screen capture images. Images from 79 % of patients were acquired as static, overlapping, single field of view studies, 15 % as whole-body only scans, and 6 % as whole-body scans with additional static single field of view images. Additional tomographic images were available in four patients, but were excluded from the analysis. Scores for the skeleton, primary tumour and soft tissues and were analysed separately.

### Assessment of skeletal tumour burden

The most common sites of skeletal metastatic involvement at baseline were femora (67 % of patients), spine (66 %), pelvis (65 %), skull (63 %), and thoracic cage (60 %), followed by the humeri (53 %) and distal lower limbs (48 %). Distal upper limb metastasis occurred less frequently (14 %).

### Correlation of initial and post-treatment skeletal score

**Fig. 3:**
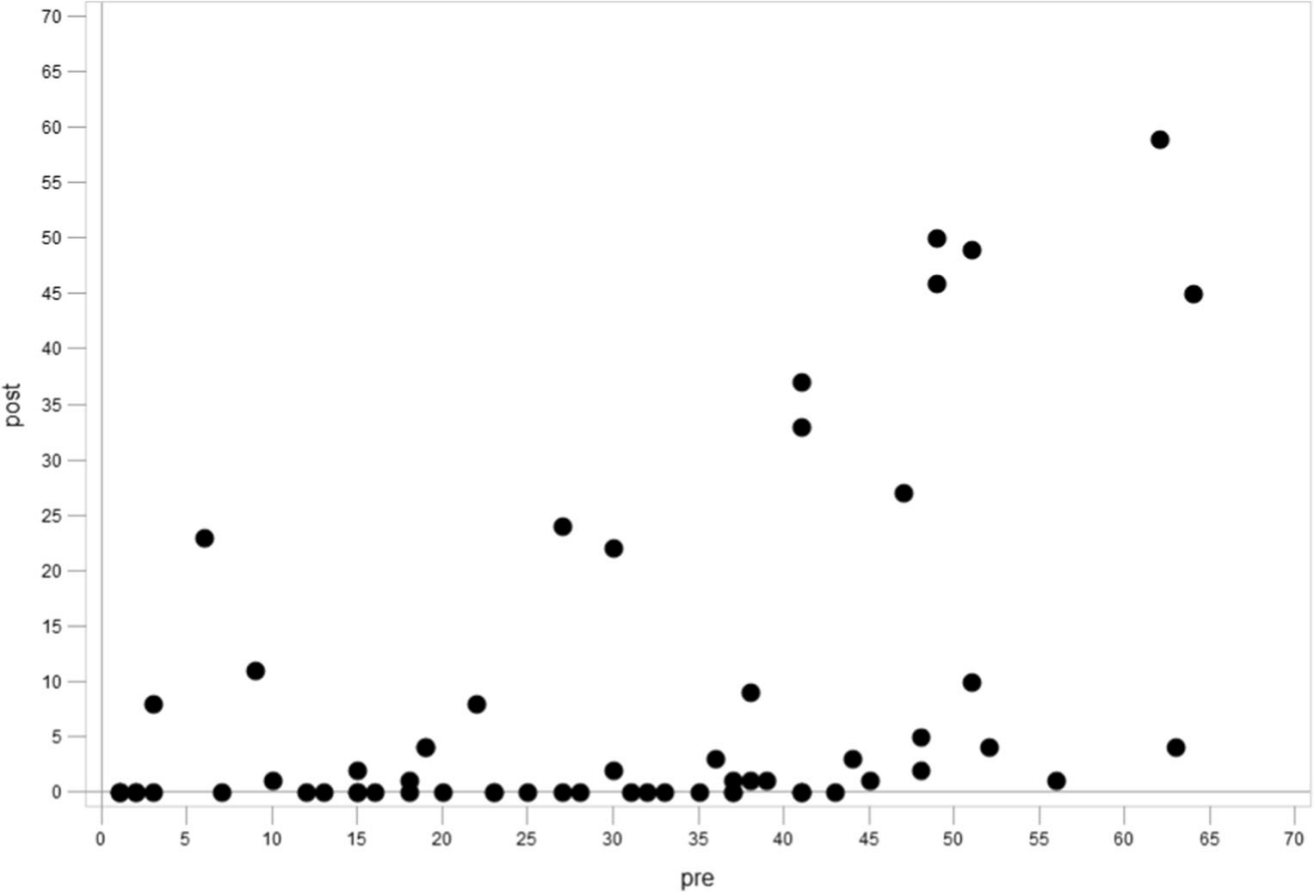
Pre-treatment skeletal mIBG score vs. post-induction chemotherapy score correlation: Spearman correlation coefficient +0.48, <0.001

A significant correlation was shown between the initial skeletal score at diagnosis and post-treatment score. Patients with extensive skeletal metastases at diagnosis had, on average, higher post induction chemotherapy scores (Spearman correlation coefficient + 0.478, < 0.001) (Fig. 3).

Patients were assigned to one of three groups according to skeletal score at diagnosis: low score (≤23) *n* = 45 (55 %); intermediate score (23 – 48) *n* = 28 (34 %); high score (>48) *n* = 9 (11 %). Skeletal response, defined as a 50 % or greater score reduction following rapid COJEC induction chemotherapy, was analysed using the Chi-squared test. Twenty patients had no skeletal mIBG uptake at diagnosis, precluding quantitative response assessment using this method. Forty-nine of 62 patients who had mIBG positive skeletal metastases at diagnosis had a 50 % or greater reduction in skeletal mIBG score following induction chemotherapy. Patients with a baseline score of >48 achieved a statistically significant poorer skeletal response rate to induction therapy (*n* = 62, *p* < 0.02). These results are summarised in Table 3.

**Table 3:** Skeletal response frequencies versus pre-treatment skeletal mIBG score

Baseline Skeletal score	<50 % post treatment score reduction		≥50 % post treatment score reduction		Total
	n	%	n	%	n
<23	3	12 %	22	88 %	25
23–48	5	18 %	23	82 %	28
>48	5	56 %	4	44 %	9
Total	13	21 %	49	79 %	62

Chi Square 7.87: *p*-value 0.020

### Skeletal disease extent scores and segment number justification

SIOPEN skeletal disease extent scores were added together to approximate a published method [3] based on a 0–3 scale as shown in Table 2.

**Fig. 4:**
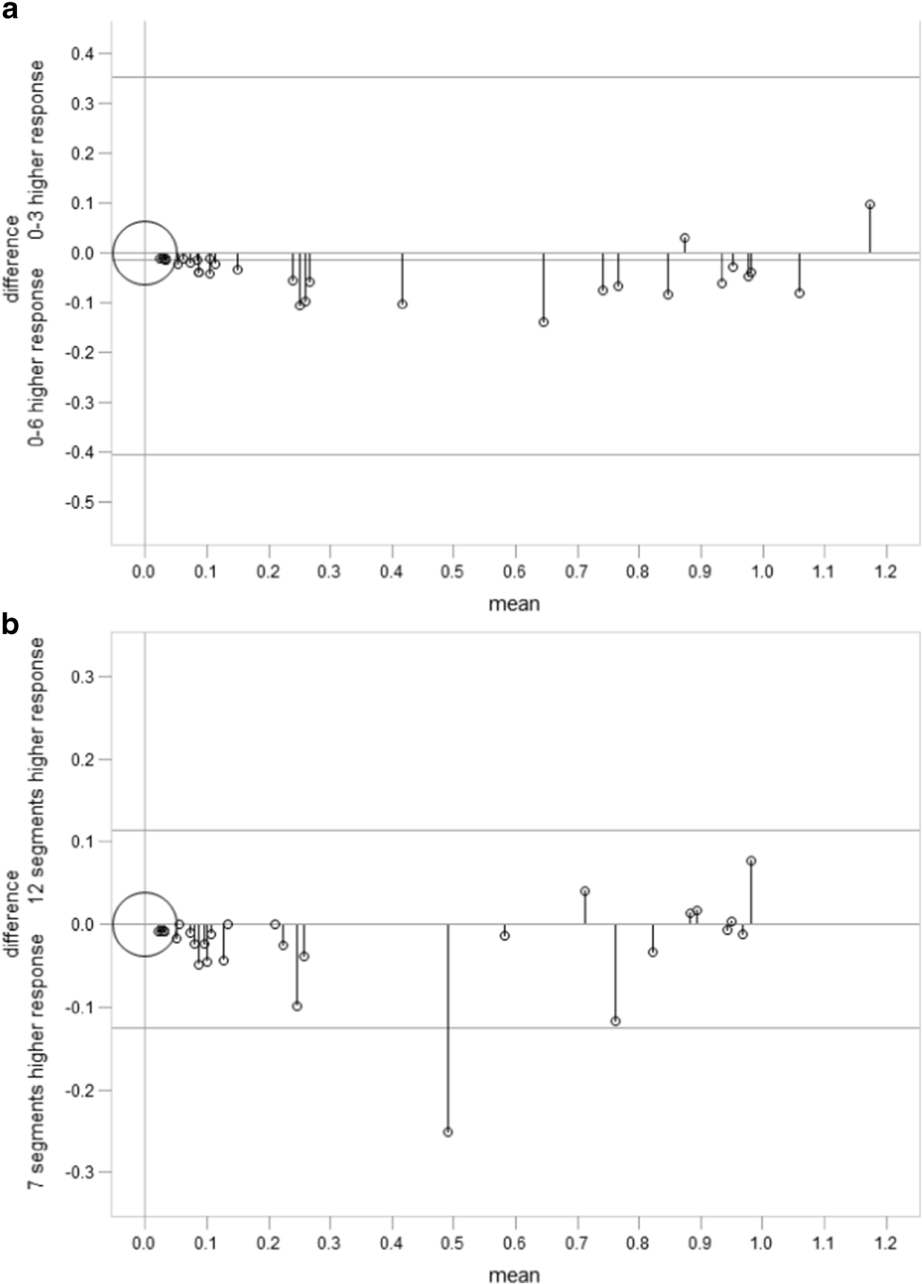
**a**. Comparison between 0–3 and 0–6 skeletal extension scales: Bland–Altman Plot: mean −0.014; standard deviation 0.19, 95 %CI -0.06-0.33 **b**. Comparison between seven and 12 skeletal segment scales: Bland–Altman Plot: mean -0.06; standard deviation 0.06, 95 %CI -0.02-0.009

The two scoring methods are compared as Bland–Altman plots in Fig. 4a. In patients with mIBG positive skeletal disease at diagnosis, a significantly higher number of favourable responses (i.e. > 50 % skeletal score reduction) was recorded using the 0–6 scale compared with the 0–3 scale (*p* < 0.001), although the mean difference between methods was small (0.03+/- 0.26).

To assess whether the method could be simplified by reducing the number of skeletal segments, skeletal disease extent scores were re-calculated based on 7 body segments as summarised in Table 1. Methods were compared using Bland and Altman plots as shown in Fig. 4b. In patients with mIBG positive skeletal disease at diagnosis, higher response rates after rapid COJEC induction chemotherapy were recorded using the 12 segment method compared with a seven-segment technique (*p* = 0.014) although the mean difference between methods was low (0.01+/-0.08).

### Inter-observer and intra-observer reproducibility and time to score

Skeletal disease extent scores were analysed to assess inter- and intra-observer reproducibility. Intraclass correlation coefficients (ICC) calculated for the unblinded study were 0.95 at diagnosis and 0.98 on completion of induction chemotherapy. Corresponding ICCs for the blinded study were 0.95 and 0.99, respectively.

The time to analyse and score an individual skeletal data set after data upload was typically 2 minutes, depending on scan complexity. The short time for analysis was facilitated by assessing images in a logical cranio-caudal anatomical sequence, separating right and left limb scores and by avoiding assessment of uptake intensity.

### Assessment of soft tissue involvement

Abnormal ^123^I-mIBG soft tissue uptake, excluding the primary tumour, was difficult to separate from skeletal and physiological activity on pre- and post-treatment planar images. Soft tissue disease was discernible in 11 patients at diagnosis and in 8 on completion of induction chemotherapy. The small sample size precluded further ICC correlation.

### Primary tumour assessment

The SIOPEN-R-NET database documented 72 abdominal, five thoracic, and five thoraco-abdominal primary tumours in the reviewed patient group. Excluding four patients who underwent primary tumour resection prior to mIBG imaging, the primary tumour was correctly identified by ^123^I-mIBG imaging in 73/78 patients at diagnosis.

Primary tumour response was recorded as qualitative size reduction by at least 50 % between pre- and post-induction chemotherapy scans. Data from 62 patients with mIBG positive primary tumours and skeletal metastases were analysed. In this cohort 22 patients (35 %) had a response of the primary tumour post induction chemotherapy. Twenty-one out of 62 responded in both the primary tumour and skeleton. One child had a 50 % primary tumour response but less than 50 % skeletal response. A significant correlation was shown between primary tumour and skeletal metastatic response as assessed by mIBG imaging (Spearman correlation coefficient +0.47, <0.001).

## Discussion

The clinical significance of standardised, objective mIBG reporting in neuroblastoma is well recognised. Earlier reporting methods for mIBG scintigraphy [13] included semi-quantitative evaluation of the intensity of mIBG uptake compared with reference normal tissue. This is feasible when images are acquired as whole-body scans in which the reference tissue (liver) is included in the data set. Uptake intensity cannot, however, be determined from single-view, static images where the reference tissue is excluded from the field of view. Although older children may be able to co-operate with lengthy whole-body scans, the majority of SIOPEN-R-NET participating trial centres prefer to acquire overlapping static images in young patients. In accordance with published guidelines [9], images are acquired either for a minimum of 600 seconds or until a pre-determined total number of counts per image has been achieved, whichever occurs earlier. Recommended total counts per image vary according to body area.

Thus, the relative intensity of ^123^I-mIBG uptake between body areas cannot be assessed from single field of view images unless every image has been acquired for an identical period of time. The strict imposition of this requirement is unworkable in paediatric oncology practice. For these reasons, we conclude that subjective intensity measurements should be excluded from semi-quantitative ^123^I-mIBG scoring methods. The new score method was developed specifically to avoid this pitfall.

Lesion intensity values have accounted for up to 60 % of total score in semi-quantitative mIBG evaluation methods published previously [3, 4]. Exclusion of intensity results would, however, lead to a very restricted score range, which might limit the sensitivity of semi-quantitative approaches for response assessment. We have overcome this both by expanding the scale used to describe tumour extent to 0–6 and by increasing the number of individual body segments evaluated to 12, generating a score range of 0–72. In the review population, this detailed evaluation allowed clear discrimination between induction chemotherapy responders and non-responders.

Data reviewed were acquired from 34 centres in 12 European countries and are considered representative of an unselected paediatric NB population. The high inter- and intra-observer ICCs achieved confirm the reproducibility of the proposed score method and that this approach is valid across a broad spectrum of high-risk NB disease. The scoring method was applied consistently by all members of the review panel who worked independently using electronically stored anonymised data. The method is, therefore, straightforward and can be applied by trained nuclear medicine specialists with minimal additional briefing. Further, the focus on skeletal extension score rather than uptake intensity reduced the time required for data review, the typical time to evaluate a single uploaded study being in the order of 2 minutes. Image scoring in a logical cranio-caudal anatomical sequence proved straightforward in practice and speed of reporting was improved by evaluating right and left limb scores separately. The method was applied intuitively by nuclear medicine specialists and facilitated peer review of large numbers of scans within a manageable time frame. As no time penalty has been encountered in undertaking the detailed segmental assessment, we suggest that this method is sufficiently promising and time-efficient to be adopted in routine practice.

In common with published experience, the extent of skeletal involvement at diagnosis correlates well with response to rapid COJEC induction chemotherapy, as assessed by post-treatment ^123^I-mIBG scans [3–5]. In our study group, children with higher skeletal scores (>48) at diagnosis had a lower chance of obtaining a 50 % reduction compared to the low (0–23) and intermediate score (24–48) groups. Establishing a score threshold that could predict both treatment response at diagnosis and outcome would allow early identification of patients suitable for dose intensification. This is a key area for future research and further studies are in progress.

Although a strong correlation was shown between response to induction chemotherapy in the primary tumour site and skeletal metastases, only 35 % of children within the review group achieved a 50 % or greater metabolic response at the primary tumour site, compared with 79 % who achieved at least 50 % reduction in skeletal mIBG score. The demonstration of differential response between metastatic and primary tumour sites in this study is consistent with clinical experience and confirms the importance of additional intervention for local disease control in high risk neuroblastoma. Although planar imaging is probably adequate to assess skeletal response, it is likely that the sensitivity of ^123^I-mIBG in evaluating primary tumours would be improved by SPECT/CT imaging. The unexpectedly low primary tumour metabolic response rate observed may, therefore, be partly attributable to a lack of tomographic information in the reviewed data set. Gamma cameras with SPECT/CT capability are becoming more widely available in Europe but longer image acquisition times and radiation dose concerns resulting from the CT component still present logistic difficulties in the paediatric population. In most centres, ^123^I-mIBG SPECT/CT is not routinely performed in young children unless uptake in overlapping tissues precludes accurate tumour definition, where this discrimination would be likely to alter management.

Evidence supporting the role of PET-tracers such as fluorine-18 deoxyglucose (FDG) and gallium-68 somatostatin analogues (DOTANOC, DOTATOC, DOTATATE) in assessing NB is emerging [13, 14] and merits further comparison with ^123^I-mIBG SPECT/CT. A validated score method for tomographic mIBG scans will be required for this purpose.

Excluding the primary tumour, soft tissue disease was unusual at diagnosis in the study population. The separate evaluation of abnormal ^123^I-mIBG soft tissue uptake, other than the primary tumour, proved very difficult on pre- and post-treatment planar images, particularly in central thoracic and abdominal compartments where osseous and soft tissue tumour uptake was often superimposed. This may be more problematic in children with relapsed NB, in whom soft tissue disease occurs more frequently. While planar scintigraphy is sufficient to document skeletal lesions, soft tissue disease is better evaluated and differentiated from physiological uptake by tomographic imaging. We, therefore, share the pragmatic view of other groups [4] that all disease should be scored within anatomical segments without differentiating between skeletal and soft tissue lesions. Given the minor contribution of soft tissue disease relative to skeletal tumour burden in the study population, this approach would have had no significant impact upon the results reported.

Although all reviewed data sets fulfilled minimum standards for completeness and specified format, a wide variation in image quality was observed. In general, image quality improved between data acquired in the early and later stages of the SIOPEN-R-NET trial. This reflects improvements both in gamma camera performance and European paediatric nuclear medicine practice that have occurred within the time frame of the high risk NB trial. Specific observations regarding mIBG imaging procedures will be reported separately.

Data sets included for review were stored electronically on the SIOPEN-R-NET database in a variety of formats. Images held in DICOM format facilitated data manipulation, contrast, and window thresholding. This increased the speed and sensitivity of image review compared with snapshot scans captured as JPEG, Bitmap, or TIFF files. We recommend that storage of image data in DICOM or Interfile format be adopted as the standard for clinical trials.

At present, ^123^I-mIBG imaging is the only widely available, objective measure of metabolic treatment response in NB. The semi-quantitative scoring method developed and described here is straightforward, scientifically robust and will enhance the reproducibility of ^123^I-mIBG image reporting in both clinical practice and clinical trials. The method would also provide a basis for comparing mIBG imaging with FDG PET CT and radiolabelled somatostatin analogues in NB.

The proposed score method draws on extensive published previous experience, but the avoidance of subjective uptake intensity assessment and detailed focus on skeletal tumour extent as a prognostic factor are new. The method has been validated in a multi-centre, international patient population and will be tested prospectively within the SIOPEN-R-NET high risk NB trial. We conclude that this approach represents a positive step in NB management, sets a new standard for response assessment and could be applied to determine the role of ^123^I-mIBG scintigraphy as an independent prognostic indicator in NB.
